# The Role of Bacterial Biofilms and Surface Components in Plant-Bacterial Associations

**DOI:** 10.3390/ijms140815838

**Published:** 2013-07-30

**Authors:** Pablo C. Bogino, María de las Mercedes Oliva, Fernando G. Sorroche, Walter Giordano

**Affiliations:** 1 Department of Molecular Biology, National University of Río Cuarto, Ruta 36 Km 601, Río Cuarto, Córdoba X5804BYA, Argentina; E-Mails: pbogino@exa.unrc.edu.ar (P.C.B.); fernando.sorroche@biol.lu.se (F.G.S.); 2 Department of Microbiology and Immunology, National University of Río Cuarto, Ruta 36 Km 601, Córdoba X5804BYA, Argentina; E-Mail: moliva@exa.unrc.edu.ar

**Keywords:** biofilms, autoaggregation, plant-associated bacteria, bacterial surface compounds, bacterial exopolymeric compounds

## Abstract

The role of bacterial surface components in combination with bacterial functional signals in the process of biofilm formation has been increasingly studied in recent years. Plants support a diverse array of bacteria on or in their roots, transport vessels, stems, and leaves. These plant-associated bacteria have important effects on plant health and productivity. Biofilm formation on plants is associated with symbiotic and pathogenic responses, but how plants regulate such associations is unclear. Certain bacteria in biofilm matrices have been found to induce plant growth and to protect plants from phytopathogens (a process termed biocontrol), whereas others are involved in pathogenesis. In this review, we systematically describe the various components and mechanisms involved in bacterial biofilm formation and attachment to plant surfaces and the relationships of these mechanisms to bacterial activity and survival.

## 1. Introduction

Biofilms are bacterial communities in which cells are embedded in a matrix of extracellular polymeric compounds attached to a surface [1]. Living in biofilms helps protect bacteria from deleterious conditions [2] and the formation of biofilms appears to be an important factor in the disease cycle of bacterial pathogens in both animals and plants.

Bacterial surface components and extracellular compounds [primarily flagella, lipopolysaccharides (LPSs), and exopolysaccharides (EPSs)], in combination with environmental and quorum-sensing signals, are crucial for autoaggregation and biofilm development in most bacterial species studied to date [3,4]. In the generally accepted model of biofilm formation, environmental signals trigger the process, and flagella are required for the biofilm community to approach and move across the surface. The initial steps of attachment are mediated by outer membrane proteins (e.g., calcium-binding proteins), pili, or LPSs. After the formation of microcolonies, the production of quorum-sensing signals is required for the formation of a mature biofilm [5]. EPSs provide the architectural form of biofilms and stabilize their 3-dimensional structure. Biofilms are often permeated by channels that act as a circulatory system, allowing the bacteria to exchange water, nutrients, enzymes, and signals, dispose of potentially toxic metabolites, and display enhanced metabolic cooperativity [4,6]. The dispersal of biofilms allows bacteria to colonize other surfaces or substrates, thus completing a sequential developmental process.

The composition of biofilms varies depending on the system. The major components are typically water and the bacterial cells, followed by the EPSs of the matrix [7], which provides (i) a physical barrier against the diffusion of antibiotics, defense substances, or other important compounds from the host; and (ii) protection against environmental stress factors, such as UV radiation, pH changes, osmotic stress, and desiccation [8,9]. In *Agrobacterium tumefaciens*, a plant pathogen that persists as surface-associated populations on plants or soil particles, cellulose overproduction resulted in increased biofilm formation on roots [10]. Minor biofilm components include macromolecules such as proteins, DNA, and various lysis products [1], which affect the overall properties of the biofilm.

Bacterial biofilms are widely distributed and play important roles in many environments. The environments occupied by soil bacteria range from rhizospheres rich in nutrients and root exudates to bulk soil deficient in nitrogen, phosphates, water, and other nutrients. The size of bacterial aggregates varies from small to large as a function of the nutrient availability at a given site [11]. A hypothetical model of various 3-dimensional shapes of root-biofilm structures determined by nutrient availability has been presented [12].

**Figure 1:**
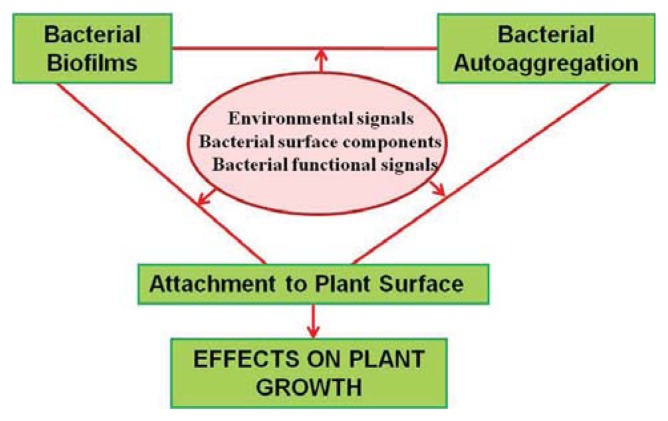
Bacterial autoaggregation and biofilm development, and their relationship with plant colonization. Cell aggregation and biofilm formation in plant-bacterial associations are regulated by environmental signals, nutrient limitation of growth, quorum sensing, EPSs, flagella, LPSs, and other factors.

Many species of beneficial soil bacteria, including rhizobia, form microcolonies or biofilms when they colonize roots. We recently summarized data on surface attachment and/or biofilm formation by rhizobacteria [5]. Biofilm development also contributes to the virulence of phytopathogenic bacteria through various mechanisms, including blockage of xylem vessels, increased resistance to plant antimicrobial compounds, and/or enhanced colonization of specific habitats [13]. The processes of autoaggregation and biofilm development are relevant to both bacterial survival and host plant colonization (Figure 1). A variety of environmental, genetic, and structural factors affect bacterial adhesion, cell-cell interactions, and plant colonization, and ultimately plant-bacterial interactions in general. In this article, we review recent findings on the mechanisms involved in attachment, cell aggregation, and biofilm formation on plant surfaces by bacteria.

## 2. Cell–Cell Adhesive Interactions: Bacterial Autoaggregation

Bacteria were studied for many years as isolated cell entities. However, like many microorganisms, they have a strong tendency to congregate or aggregate. A common phenotypic manifestation of this behavior is autoaggregation, which is based on adhesive interactions among bacteria. Autoaggregation can be visualized macroscopically by the typical clumping or “fluffing” of cells in liquid cultures, followed by sedimentation of the clumps under static conditions [3,14,15].

Unfavorable growth conditions or low metabolic activity have been found to induce aggregative behavior in bacteria that normally grow in a dispersed, non-aggregated manner. In this context, autoaggregation may reflect a survival strategy that is triggered under hostile environmental conditions [16–19].

The autoaggregative characteristic of bacteria has important implications for the production of bacteria-based inoculants for agriculture. Bacterial aggregates can be produced on a large scale and then separated more easily from the culture medium as compared with dispersed bacteria. The biomass of aggregated bacterial cells in bioreactors remains more constant, and the survival of such bacteria during the inoculant storage period is enhanced, in comparison with non-aggregated cells [17,18,20,21].

### 2.1. Surface Factors Involved in Bacterial Autoaggregation

Because of their strategic location on the cell surface, LPSs, outer membrane proteins, and proteinaceous structures such as pili have been reported to affect adhesion among bacteria and consequently the autoaggregation phenotype.

LPS is an important surface structural component of Gram-negative bacteria and covers ~75% of the surface area of the outer membrane. It is a tripartite molecule consisting of lipid A, core oligosaccharides, and *O*-antigen, structurally formed by amphiphilic glycoconjugates whose composition varies within and between species. LPS molecules are positioned among the proteins and phospholipids of the outer bacterial membrane and contribute to the structural properties of the membrane; e.g., they act as a permeability barrier against various types of molecules. The structural heterogeneity of the *O*-antigen, the most external portion of the LPS molecule, confers versatility and adaptability to bacteria that are exposed to variable environmental conditions [22]. Changes of LPS structure usually affect adhesive forces among bacteria, possibly through alteration of cell surface hydrophobicity. For example, in rhizobacteria such as *Rhizobium leguminosarum* and *R. etli*, LPS modifications typically alter the autoaggregation phenotype [23–25]. *Ensifer* lpsB mutants, which have a truncated LPS core, display a more strongly autoaggregative phenotype as compared with wild-type parental strains (Sorroche *et al*., unpubl. data). The rhamnose-rich *O*-antigen in the outermost part of the LPS of the xylem-limited phytopathogen *Xylella fastidiosa* is involved in cell-cell aggregation [26]. The autoaggregative ability of *X. fastidiosa* appears to be an important virulence mechanism because the bacterial clusters block the passage of water and nutrients from the roots to the leaves of the host plant [27–29].

Bioassay studies of the impact of biological factors on *Azospirillum brasilense* autoaggregation indicated that outer membrane proteins promote bacterial flocculation [30]. A 67-kDa outer membrane lectin on the bacterial surface specifically recognized an EPS synthesized by the aggregated cells. The interaction between the lectin and the EPS may be responsible in part for cell-cell interactions leading to autoaggregation of this species [31].

Pili, fimbriae, and flagella are proteinaceous polymeric appendages acting as bacterial surface organelles. Their numerous functions include mediation of motility, interbacterial interactions, bacterial-host interactions, and surface colonization [32]. Pili are associated with autoaggregation in *X. fastidiosa*. This process depends on the presence of polarly located type I and type IV pili, each of which plays a specific role in the structural dynamics of the bacterial aggregates [33].

### 2.2. Extracellular Factors Involved in Bacterial Autoaggregation

Some bacteria secrete molecules that promote autoaggregation. Extracellular polymeric materials have been shown to act as “molecular glue” that initiates and maintains contact between cells, causing flocculation. The main extracellular compounds are EPSs, which are linear or branched molecules formed by one repeated sugar (homopolysaccharides) or by a mixture of different sugars (heteropolysaccharides). An example is galactoglucan (EPS II) from the symbiotic rhizobacterium *Ensifer meliloti*. This extracellular EPS is secreted in two major fractions, low molecular weight (LMW) and high molecular weight (HMW), according to the degree of polymerization [34]. Mutant strains that are unable to synthesize EPS II fail to autoaggregate under static conditions. A mucR mutant secreting almost exclusively the HMW fraction of EPS II showed a weak aggregative phenotype, suggesting that the LMW fraction plays the active role in autoaggregation. Aggregation of the non-EPS-producing strains and the mucR strain was restored by resuspending the cells in culture medium containing EPS II [14].

Cellulose is an exopolymer with agglutinating activity in *R. leguminosarum*. Upon contact with the host plant, this rhizobacterium aggregates on the root surface using cellulose microfibrils [35–37].

Hostile environmental conditions or low metabolic activity can induce an autoaggregative phenotype via the synthesis of EPSs that have agglutinating activity. For example, the aggregative phenotype of the rhizobacterium *A. brasilense* depends on the production of an arabinose-rich extracellular polysaccharide that is synthesized in cultures in stationary and programmed cell death phases. Aggregative behavior in *Pseudomonas aeruginosa* is triggered by the presence of the toxic detergent SDS. This behavior depends on production of the EPS PsI. Inactivation of either the psi gene or the c-di-GMP-mediated signaling system that activates the gene results in reduced autoaggregation [19,38,39].

## 3. Cell–Cell and Cell–Surface Interactions: Bacterial Biofilm Formation

Surface and extracellular bacterial components have been extensively studied because they involve molecules that play crucial roles during the process of infection of the host plant, independently of the development of a beneficial or pathogenic relationship. Such bacterial components are also key molecules in the establishment, maturation, and dispersal of biofilms. We will summarize in this section the structure and function of bacterial compounds that play a role in the development of biofilms by beneficial or pathogenic bacteria associated with the surface or interior of plant tissues.

### 3.1. Structural and Functional Components Involved in Biofilm Formation

All bacteria live as a multicellular conglomerate encased in a protective matrix of polymeric substances produced by the bacteria themselves. The highly organized and dynamic social structure of bacteria requires intercellular communication via quorum sensing [40]. Bacterial aggregates frequently adhere at a solid-liquid interface prior to adsorption on a thin film of organic molecules that constitutes the adhesion site. The transport of cells to this interface may be mediated by passive mechanisms or by the intrinsic motility of planktonic bacteria. The accumulation of bacterial cells at the interface is biphasic, consisting of (i) a non-specific reversible stage mediated by hydrophobic and electrostatic interactions between cells and adjacent surfaces; and (ii) an irreversible stage in which the adhesion process is completed and a bacterial microcolony is established [41]. Surface bacterial components such as flagella, pili, fimbriae, and LPSs play a crucial role in physical processes during the initial stages of biofilm formation on surfaces. The growth, maturation, and disassembly phases of biofilms depend primarily on the biosynthesis of extracellular biopolymers such as EPSs, proteins, and extracellular DNA (eDNA). These polymers promote or provide immobilization of bacterial cells into the matrix, mechanical stability of the biofilm structure, cohesive interaction with the interface, and the architecture and functionality of the encased microbial community [42].

#### 3.1.1. Surface Bacterial Factors

Because of their exposure to the external environment and their chemical properties, LPSs on the outer membrane of Gram-negative bacteria are capable of undergoing adhesive interactions with both biotic and abiotic surfaces.

Structural changes of LPSs have been shown to alter biofilm formation or structure in beneficial plant-associated bacteria, including *Pseudomonas fluorescens* [43]. Mutant strains of various beneficial rhizobacteria with altered LPS structure display changes in the biofilm formation process. For example, a *Bradyrhizobium japonicum O*-antigen mutant showed enhanced adhesion to plastic supports [44]. A *R. leguminosarum* lipid a mutant showed increased lateral interactions with an abiotic surface; this effect had no effect on the ability of the bacteria to form a biofilm on the surface [45]. A mutant of the alfalfa symbiont *E. meliloti* that synthesized a structurally modified LPS because of mutations of the *lpsB* and *bacA* genes showed reduced biofilm formation ability [46,47]. *E. meliloti lpsB* mutants showed reduced nodulation abilities because of delays in the invasion steps; however, their nitrogen-fixing capacity was similar to that of wild-type [48,49], and the modified LPS molecule increased bacterial adsorption to alfalfa roots [50].

In phytopathogenic bacteria, as in beneficial bacteria, LPSs play crucial roles during the early stages of interaction with the host and development of virulence. These phenomena coincide with the normal stages of biofilm formation. Various mutations related to LPS synthesis in phytopathogenic bacteria such as *P. aeruginosa* [51], *Pseudomonas syringae* [52], *Xanthomonas axonopodis* [53], and *Xanthomonas citri* [54] caused reductions in both biofilm formation ability and virulence.

Because of their surface cell location and physico-chemical properties, LPSs play a key role during the initial steps of biofilm formation (e.g., adherence to surfaces) and the development of mature biofilm through interactions of cells with other cells and with matrix components.

Proteinaceous appendages (pili and flagella) are bacterial virulence factors that lead to pathogenesis in plant, animal, and human hosts and play a key role during colonization steps [55]. The initial stages of biofilm formation are dependent on bacterial motility mediated by the polar flagellum and multiple type IV pili (TfP), which enable the free-swimming phenotype to reach a suitable surface and the surface-motile phenotype to adhere to and move on the surface [56]. These appendages thus have a dual role as motile machines and adhesins that move and fix bacteria to surfaces and among surfaces [57]. Various types of movement (e.g., crawling, pulling, walking) have been associated with TfP [56]. The coordinated TfP pulling associated with release and with flagellar rotation-translation has been presented as a model whereby bacteria are able to travel through the biofilm matrix [58]. Motility-defective mutants of *P. aeruginosa* and *E. coli* were unable to attach to surfaces or to develop a normal biofilm [59,60].

Pili structure is crucial for adhesion and biofilm formation in certain phytopathogenic bacteria, including *Acidovorax citrulli*, the causal agent of bacterial fruit blotch in cucurbits [61], and *X. fastidiosa*, the causal agent of Pierce’s disease in grapes [62]. *A. citrulli* uses TfP and *X. fastidiosa* uses type I pili to colonize and move upstream against sap flow in xylem vessels while oriented parallel to the surface, prior to the complete development of biofilm and plant disease. TfP is also important for the pathogenesis of *Ralstonia solanacearum* and *Xanthomonas oryzae* pv. *oryzicola* [63,64]. Pili are well-established virulence factors for phytopathogenic bacteria because of their important roles in adherence and plant colonization.

Motility mediated by flagella is manifested as either “swimming” of free cells in aqueous environments and coordinated “swarming” of bacterial populations on solid moist surfaces [65]. Both swimming and swarming are essential for various stages of biofilm development, e.g., the search for a favorable habitat, attachment to a surface, architectural assembly, structural disassembly, and release from the biofilm matrix [66]. The complex association between motility and biofilm formation involves the use of a particular structure for different functions at different stages and requires the precise integration of environmental and cellular signals [67].

Flagella-mediated motility in rhizobacteria is essential for biofilm establishment and therefore for plant colonization. *E. meliloti*, a paradigm of beneficial symbiotic interaction between rhizobacteria and legume plants, showed reduced biofilm formation ability on abiotic surfaces as a result of mutations on genes related to flagella synthesis [68]. An association between flagella-mediated motility and biofilm formation and an effect of quorum-sensing signals on both of these processes were demonstrated in peanut-nodulating *Bradyrhizobium* sp. strains [69]. Both aflagellate and flagellated but nonmotile mutants of the well-studied pathogen *A. tumefaciens*, the causal agent of crown gall, showed reduced biofilm formation ability under static conditions because of defects in surface attachment. However, the aflagellate mutants were able to quickly develop an unusually dense and tall biofilm under flow conditions [70]. In contrast to results of other studies that suggest a role of flagella as an adhesin in *Aeromonas* spp. [71], these results for *A. tumefaciens* are consistent with those for other bacterial species [72], which suggest that flagella do not function as an adhesin and that other surface structures can be involved in attachment and subsequent biofilm formation [70].

*X. axonopodis* pv. *citri*, a phytopathogenic bacterium that establishes itself on the leaves (phyllosphere) of citrus species and produces citrus canker disease [73], has the ability to form biofilms on abiotic and biotic surfaces, including the cankers of diseased plants [74]. Flagella-mediated motility plays a key role in several stages of biofilm formation, including surface adherence, maturation, and dispersal [75]. Sliding motility (not mediated by flagella) and the regulation of both swimming and sliding motility through diffusible signal factor (DSF) were reported for *X. axonopodis* [75]. Similar findings were reported for *Xanthomonas campestris* pv. *campestris*, although flagellin mutants did not show altered virulence [76]. Swimming motility was found to be essential for biofilm formation and colonization of plant tissues in vascular pathogens such as *R. solanacearum*, *Pantoea stewartii*, and *Dickeya dadantii* [77–79].

In contrast to findings in Gram-negative bacteria, it appears that motility is not essential for biofilm development in various non-motile Gram-positive bacteria. Surface proteins (e.g., Bap, Esp) have been reported to be involved in initial adherence to surfaces in these non-motile species [80,81].

Several surface structures that function as adhesins have been found to play important roles during surface attachment. Rhicadhesins and Raps (*Rhizobium-*adhering proteins) are important for root attachment in various Rhizobiaceae species [82,83]. Glucomannan, a surface polysaccharide of *R. leguminosarum* bv. *trifolli*, binds to pea and vetch lectins [84]. Two surface-associated proteins were found to be involved in biofilm formation in *Pseudomonas putida*. LapA plays a key role in the early stages of biofilm formation by mediating bacterial adherence to various surfaces (including seeds and roots), while LapF is crucial in later stages by mediating cell-cell interactions during sessile growth [85,86].

#### 3.1.2. Extracellular Factors

The highly developed level of bacterial organization reflected by biofilm formation ability and multicellularity requires dynamic and functional microorganisms that are embedded into a complex mixture of extracellular polymeric components collectively termed the “biofilm matrix” [1]. These components form bridges, channels, avenues, and pores and support an impressively elaborate 3-dimensional architectural structure within which cellular arrangements are transiently constructed. In addition to its structural protective function, the biofilm matrix plays a key role in bacterial physiology and ecology, including cellular interactions, nutrient utilization, horizontal gene transfer, and environmental fitness of the bacterial population [42]. The composition of the biofilm matrix is highly variable, depending on the type of bacteria and biofilm interface. Water is the main component of a mature biofilm. In terms of dry mass, bacteria account for <10% of a biofilm, and the matrix accounts for >90%. The biofilm matrix consists primarily of EPSs and contains smaller proportions of other biopolymers such as proteins, nucleic acids, and lipids [7]. We will summarize here the roles of the major components of the biofilm matrix; *i.e.*, extracellular factors.

A biofilm has been defined as a multicellular bacterial conglomerate adhered to a surface and immersed into a polymeric matrix formed primarily by EPSs [4]. It is therefore not surprising that various bacterial strains, even of the same species, have the ability to synthesize, export, and modify their own characteristic EPS. The function and chemical composition of each EPS is different, depending on the bacterial species or strain. A particular strain may even have the ability to produce different EPSs depending on the environmental conditions, as demonstrated for *P. aeruginosa* [87] and *Streptococcus thermophilus* [88]. Most EPSs are polyanionic molecules because of the presence of uronic acids and sugar having substituents such as pyruvate, sulfate, or phosphate. Polycationic EPSs have also been described [89]. The presence of β-1,4 (or β-1,3) and α-1,2 (or α-1,6) linkages confers greater rigidity or flexibility, respectively, to the matrix structure. The stability of the biofilm structure is thus dependent on the physico-chemical and biological properties of EPSs and on their interactions with ions, low molecular weight solutes, and other macromolecules such as proteins and eDNA [7]. EPSs are generally required not for initial adhesion but for later architectural development of the biofilm matrix [90]. The EPS network confers mechanical stability, allows for temporary immobilization of cells, and plays a crucial role in most matrix functions, including water retention, protection from environmental stresses, adsorption of compounds, and nutrient availability [42].

The production of EPSs on plant surfaces or tissues allows bacterial colonization and biofilm formation. The biological roles (beneficial or pathogenic) of bacteria on plants are related to these abilities. The compositions and biological roles of selected EPSs produced by well-known plant-associated bacteria are summarized in Table 1.

The importance of cellulose (a neutral homopolysaccharide) as a key component of the polysaccharidic matrix has been demonstrated in several Enterobacteriaceae species [118], the plant-associated bacterium *A. tumefaciens* [119], and *Rhizobium* species [36,120]. Cellulose plays a key role in adherence to plant tissues, biofilm formation, and the support of matrix architecture.

Polysaccharides from *Arabidopsis* roots were recently found to serve as both signals for biofilm formation and a source of sugars for the synthesis of matrix EPSs in the beneficial Gram-positive bacterium *Bacillus subtilis* [121]. Future studies on the triggering of bacterial biofilm formation by plant root exudates will be useful.

Extracellular proteins are major constituents of the biofilm matrix, but have received relatively little study in comparison with other components such as EPSs. Proteins in the biofilm matrix have both structural and physiological functions. Some matrix proteins function as extracellular enzymes and are associated with activities such as the degradation and recycling of biopolymers for nutrient availability and the modification of other exopolymers for shaping or releasing of cells from the biofilm structure. Enzymes that play such roles in the biofilm matrix include lipases, hydrolases, lyases, and glycanases [83,122,123]. Certain enzymes released by pathogenic bacteria may act as virulence factors [124,125], but such a function has not been evaluated in the context of biofilms on plant surfaces.

Some proteins in the biofilm matrix have structural functions, e.g., as lectins that bind bacterial cells to the polymeric matrix. Examples of such extracellular carbohydrate-binding proteins include a glucan-binding protein in *Streptococcus mutans* [126], LecA and LecB in *P. aeruginosa* [127,128], TasA in *Bacillus subtilis* [129], and lectins in *A. brasilense* [31]. In *P. aeruginosa*, a large quantity of matrix proteins was found in outer membrane vesicles, a typical matrix biofilm component in this species [130]. Amyloids are another common type of matrix protein with extracellular adhesin function [131].

Biofilms provide an ideal location for the exchange of genetic material. Higher levels of conjugation have been demonstrated for bacterial populations in biofilms as compared with planktonic bacteria [132]. eDNA is an important constituent of the biofilm matrix [133] and plays a role in biofilm formation in various bacterial species, including *P. aeruginosa* [134] and *Bacillus cereus* [135]. The quantity, localization, and origin of eDNA vary depending on the bacterial species. Some studies found that eDNA is arranged in certain patterns [136] and that its release is based on the lysis of certain types of bacteria [137], suggesting the occurrence of programmed cell death in biofilms [138]. In Gram-positive bacteria, eDNA is involved in adhesion to hydrophobic surfaces and in bacterial autoaggregation [139].

Lipids are also components of the biofilm matrix [140], although they have received little study in the context of plant-bacterial associations. Lipids in biofilms generally act as biosurfactants with functions such as surface activity, dispersal and bioavailability of hydrophobic substances, antibacterial or antifungal properties, and bacterial attachment and detachment [141]. Such properties have been well characterized for the rhamnolipids of *P. aeruginosa*, which play important roles at several stages of biofilm development, including surface interaction, microcolony formation, structural maintenance, and biofilm dispersal [142].

Our knowledge of the identification and functions of extracellular proteins, eDNA, and lipids in the biofilm matrix of plant-associated bacteria remains limited and fragmentary. Further studies along this line will greatly enhance our understanding of the process of biofilm formation.

## 4. Relationship between Biofilm Formation and Bacterial Autoaggregation

Bacteria have the unique ability to form complex cellular assemblies on biotic surfaces (animal tissues, plant tissues, detritus) and abiotic surfaces (sediments, soil particles, medical or laboratory instruments). Depending on the quantities of cells and extracellular components, such assemblies range from random cell aggregations observed on surfaces or in liquid suspension to complex, highly developed assemblies of cells encased in a exopolymeric matrix and attached to a surface (*i.e.*, biofilms) [143]. There are several bacterial models that are useful for studying the relationships between cell aggregations and biofilms. The adhesin AIDA, a surface glycoprotein of *E. coli*, has been shown to promote strong bacterial autoaggregation, biofilm formation, and *in vitro* adhesion to human and mammalian cells [144]. Strains of the opportunistic bacterium *Myroides odoratus* that showed strong adhesion to inert supports also showed enhanced autoaggregative ability [145]. Changes in the cell surface of *Porphyromonas gingivalis* (a pathogenic bacterium involved in periodontal disease) led to increased cell-cell interactions and consequent increases in autoaggregation and biofilm formation [146].

Variants of the phytopathogen *X. fastidiosa* with mutation of a gene involved in signal transduction showed a modified transcriptional profile of genes related to biofilm formation and bacterial aggregation traits such as surface attachment, EPS synthesis, and virulence [147].

Autoaggregative behavior has also been correlated with biofilm formation ability in beneficial plant-associated bacteria. Strongly autoaggregative mutants of the rhizobacterium *A. brasilense* showed a high tendency to form biofilms on inert supports [148]. In the rhizobacterium *E. meliloti*, autoaggregation [14] and biofilm formation [5] depend on a combination of bacterial signals, surface components, and EPSs. The development of cell interactions in both sessile populations and planktonic aggregations of various native strains of *E. meliloti* showed a positive correlation with the above processes and a requirement for EPS II, indicating the involvement of the same physical adhesive forces in autoaggregation and biofilm formation [15].

Surface and extracellular factors are involved in both cell aggregation and biofilm formation, processes that depend in part on physical interactions among bacteria. It is therefore reasonable to presume that alterations in one of these processes lead to changes in the other. Cell aggregation on surfaces most likely represents a transitional state that precedes the development of a structured biofilm. We postulate that the linkage of or transition from bacterial aggregation to biofilm formation is crucial for the establishment of beneficial or pathogenic relationships between bacteria and plants. Such relationships are developed through (i) interactions among bacteria in or near the plant microenvironment (e.g., rhizosphere or phyllosphere); (ii) interactions between bacteria and plant surfaces (e.g., leaf or root epidermis, root hairs, transport vessels); and (iii) biofilm formation and their biological effects.

## 5. Intergeneric Adhesive Interactions: Coaggregation

The coaggregation process has been defined as adhesion among genetically different microorganisms [149]. The phenomenon of coaggregation was initially described for bacteria that inhabit the human oral cavity and subsequently extended to bacteria found in other habitats, including aquatic environments and sludge [150]. Coaggregation processes and factors that affect them have been described for plant growth-promoting rhizobacteria (PGPR) [151]. These findings are relevant to our understanding of plant-bacterial interactions and the development of commercial inoculants.

Coaggregation is an integral process in the formation of mixed biofilms and is therefore ecologically important. Two models have been proposed to explain how a mixed biofilm can support different types of planktonic phase-derived bacteria. According to the first model, planktonic free cells in suspension specifically recognize and adhere to genetically different bacteria in a biofilm. The second model involves initial coaggregation of planktonic bacteria and subsequent adhesion and integration of the coaggregate during biofilm formation [149]. Regardless of these models, the coaggregation phenomenon was found to be dependent on cell surface hydrophobicity and the partner strains that participated in the interaction [152].

On a molecular level, coaggregation of human intestinal or oral bacteria and of aquatic bacteria depends on the interaction of a lectin of one participant with a complementary glycosidic receptor of the other participant [153]. Similar mechanisms are expected in bacteria that establish associations with plants, particularly in certain habitats in which bacterial populations are highly diverse, e.g., rhizospheric soil. Cell-free culture supernatants of *E. meliloti* containing EPS II induced the autoaggregation of several rhizospheric bacteria, including strains of *Pseudomonas*, *Azospirillum*, and *Burkholderia*. These findings suggest that interactions between EPS II and rhizobacteria play an important ecological role. EPS II itself may be capable of connecting different bacterial cells. According to this hypothetical model, EPS II-producing *E. meliloti* cells may act as “bridges” during the process of coaggregation with other rhizospheric bacteria [50].

## 6. Concluding Remarks

The bacterial cell surface plays a key role in bacterial aggregation, which in turn promotes bacterial dispersal, survival, and the ability to adhere to plant surfaces. It has been well documented that bacterial autoaggregation and biofilm development, and the relationship of these processes with plant colonization, are dependent on both surface bacterial factors and extracellular factors.

Bacteria gain several advantages from living in biofilms, including protection from predation, desiccation, and exposure to antibacterial substances, and improved acquisition of nutrients released in the plant environment. Biofilms provide survival sites for both beneficial and opportunistic pathogenic bacteria, by providing protection as above and increasing the potential of the bacteria to survive and evolve in the plant environment. Biofilms have been shown to enhance (i) the fitness of individual bacteria and (ii) more generalized plant health and productivity as a result of the cumulative selective advantage of the individual bacteria.

Detailed elucidation of the mechanisms involved in the various stages of biofilm formation will improve our understanding of microbial adaptations to this mode of life in a wide range of environments and of the interactions of bacteria with their eukaryotic hosts. Multidisciplinary studies using new approaches will clarify the ways in which bacteria move and interact in a variety of surface microenvironments during biofilm development. Such knowledge will enhance our understanding of biofilm formation on plant surfaces and of the sophisticated processes of interaction between prokaryotes and eukaryotes.

## Figures and Tables

**Table 1:** Production, composition, and functional roles of exopolysaccharides (EPSs) in beneficial and pathogenic plant-associated bacteria.

Bacteria/plant association	Exopolysaccharide	Chemical composition	Function
*E. meliloti* symbiosis with *Medicago sativa*	Succinoglycan (EPS I), LMW-HMW [91]	Octasaccharide units (glucose:galactose 7:1, bearing succinyl, acetyl, and pyruvyl substituents)	Required for biofilm formation [68] EPS I LMW symbiotically active [92]
	Galactoglucan (EPS II), LMW-HMW [93]	Disaccharide units (acetylated glucose-pyruvylated galactose)	EPS II LMW symbiotically active [34] EPS II LMW controls biofilm formation [94]
*R. leguminosarum* symbiosis with *Trifolium*, *Pisum*, *Vicia* and *Phaseolus* spp.	Acidic EPS [95,96]	Octasaccharide units (glucose:glucuronic acid:galactose 5:2:1, modified by acetyl, pyruvyl and 3-hydroxybutanoyl groups)	Development of a structured biofilm [83,97] Required for infection and nodulation [98,99]
*B. japonicum* symbiosis with *Glycine max*	EPS [100,101]	Pentasaccharide units (mannose:galacturonic acid:glucose:galactose 1:1:2:1)	Biofilm formation on both inert and biotic surfaces. Roles during the early stages of interaction with the host plant (initial attachment of rhizobia to root epidermal cells) [102]
*M. tianshanense* symbiosis with *Glycyrrhiza uralensis*	EPS	ND	Involved in biofilm formation and successful establishment of symbiosis [103]
*A. tumefaciens* ubiquitous plant pathogen	Succinoglycan [104]	See above	Increased production of succinoglycan results in reduced attachment and biofilm formation [105]
*X. fastidiosa* plant pathogen	Putative Fastidian gum [106]	Putative tetrasaccharide units (glucose-1-phosphate, glucose, mannose, and glucuronic acid)	Possibly involved in bacterial pathogenicity [106] Cell attachment and overall biofilm formation [107]
*X. campestris X. axonopodis* plant pathogens	Xanthan gum [108]	Pentasaccharide units (glucose:mannose:glucuronic acid 2:2:1 derivatized with acetyl and pyruvyl moieties)	Essential for microcolony formation [74] Formation of structured biofilms on abiotic surfaces and in infected plants [109,110]
*P. stewartii* plant pathogen	Stewartan [111]	Heptasaccharide units (glucose:galactose:glucuronic acid 3:3:1)	Essential for appropriate adhesion and for maturation of biofilm structure. Also a virulence factor required for effective host colonization and efficient dissemination through xylem vessels [112]
*E. amylovora* plant pathogen	Amylovoran [113]	Pentasaccharide units (galactose:glucose 4:1, and pyruvate residues)	Pathogenicity factor required for biofilm formation [114]
	Levan [115]	Homopolymer of fructose	Virulence factor. Also contributes to biofilm formation [114]
*R. solanacearum* plant pathogen	Acidic EPS I [116]	Putative structure composed by *N*-acetylgalactosamine and amino sugars (bacillosamine, galactosaminuronic acid)	Major virulence factor [117]

LMW: low molecular weight; HMW: high molecular weight; ND: not determined.
